# NMR Detection of Semi-Specific Antibody Interactions in Serum Environments

**DOI:** 10.3390/molecules22101619

**Published:** 2017-09-27

**Authors:** Saeko Yanaka, Toshio Yamazaki, Rina Yogo, Masanori Noda, Susumu Uchiyama, Hirokazu Yagi, Koichi Kato

**Affiliations:** 1 Institute for Molecular Science and Okazaki Institute for Integrative Bioscience, National Institutes of Natural Sciences, 5-1 Higashiyama, Myodaiji, Okazaki 444-8787, Japan; saeko-yanaka@ims.ac.jp (S.Y.); yogo@ims.ac.jp (R.Y.); 2 Graduate School of Pharmaceutical Sciences, Nagoya City University, 3-1 Tanabe-dori, Mizuho-ku, Nagoya 467-8603, Japan; hyagi@phar.nagoya-cu.ac.jp; 3 NMR Facility, Division of Structural and Synthetic Biology, Center for Life Science Technologies, RIKEN, 1-7-22 Suehiro-cho, Tsurumi-ku, Yokohama City, Kanagawa 230-0045, Japan; toshio.yamazaki@riken.jp; 4 Graduate School of Engineering, Osaka University, 2-1 Yamadaoka, Suita, Osaka, 565-0871 Japan; masanori.noda@u-medico.co.jp (M.N.); suchi@bio.eng.osaka-u.ac.jp (S.U.); 5 U-Medico Inc., 2-1 Yamadaoka, Suita, Osaka 565-0871, Japan

**Keywords:** Fc, NMR spectroscopy, polyclonal antibody, serum, stable isotope labeling

## Abstract

Although antibody functions are executed in heterogeneous blood streams characterized by molecular crowding and promiscuous intermolecular interaction, detailed structural characterizations of antibody interactions have thus far been performed under homogeneous in vitro conditions. NMR spectroscopy potentially has the ability to study protein structures in heterogeneous environments, assuming that the target protein can be labeled with NMR-active isotopes. Based on our successful development of isotope labeling of antibody glycoproteins, here we apply NMR spectroscopy to characterize antibody interactions in heterogeneous extracellular environments using mouse IgG-Fc as a test molecule. In human serum, many of the HSQC peaks originating from the Fc backbone exhibited attenuation in intensity of various magnitudes. Similar spectral changes were induced by the Fab fragment of polyclonal IgG isolated from the serum, but not by serum albumin, indicating that a subset of antibodies reactive with mouse IgG-Fc exists in human serum without preimmunization. The *metaepitopes* recognized by serum polyclonal IgG cover the entire molecular surface of Fc, including the binding sites to Fc receptors and C1q. *In-serum* NMR observation will offer useful tools for the detailed characterization of biopharamaceuticals, including therapeutic antibodies in physiologically relevant heterogeneous environments, also giving deeper insight into molecular recognition by polyclonal antibodies in the immune system.

## 1. Introduction

Most biopharmaceuticals, including therapeutic antibodies, function in the blood stream, which is a considerably heterogeneous environment characterized by molecular crowding and promiscuous intermolecular interactions. Antibodies are a major class of serum protein, and are potentially involved in specific and non-specific interaction networks in physiological environments, as best exemplified by the idiotype network [1]. Furthermore, it has been proposed that serum immunoglobulin (Ig) can be reactive with endogenous lectins through carbohydrate moieties attached to the Fc region under pathological conditions [2]. Moreover, a recent analytical ultracentrifugation study reported that serum albumin interacts with IgGs and affects the sizes of immune complexes [3].

A variety of engineered Igs and Ig-derivatives are currently being developed for therapeutic and diagnostic applications [4]. These non-self proteins may be recognized as antigens by the immune system. In particular, the elicitation of human antibodies directed against heterologous Ig-based molecules used for therapy or diagnostic imaging has been a significant problem [5]. Furthermore, humanized IgG preparations have also been reported to cause undesirable infusion reactions [6,7]. Thus, detailed characterization of serum protein interactions is desirable for the development of biopharmaceuticals with minimal side effects. However, detailed structural characterizations of antibody interactions have thus been performed almost exclusively under homogeneous in vitro conditions, employing crystallographic and spectroscopic techniques [8,9,10].

NMR spectroscopy is a potentially powerful tool for studying protein structures and interactions at the atomic level, even in heterogeneous environments. This has been demonstrated with recently emerging in-cell NMR approaches, which has underscored the different structural and interaction properties of proteins in the intracellular milieu in comparison to in vitro conditions [11,12]. In such NMR applications, labeling of the target protein with NMR-active stable isotopes, e.g., ^15^N, is necessary for selectively observing the target against the background proteins. In contrast to intracellular proteins, proteins in the extracellular environment, typified by antibodies, are secreted mostly with glycosylation, with the notable exception of serum albumin [13]. The carbohydrate moieties affect not only the physical properties of secretory glycoproteins as biopharmaceuticals, such as solubility and thermostability, but also their biological properties, including serum half-life and functional protein–protein interactions, as well as antigenicity [14,15,16]. Although stable isotope labeling of such glycoproteins has been difficult to achieve, we have successfully developed a method to accomplish this by employing a variety of eukaryotic expression systems using monoclonal antibodies as model glycoproteins [17,18,19,20,21,22].

This success has prompted us to apply NMR spectroscopy to study structures and interactions of recombinant glycoproteins in extracellular heterogeneous environments, such as in blood. As the first step, in the present study, we attempt to examine the applicability of NMR to observe a specific glycoprotein in a serum environment using an Fc fragment of mouse IgG as the test molecule.

## 2. Results and Discussion

**Figure 1:**
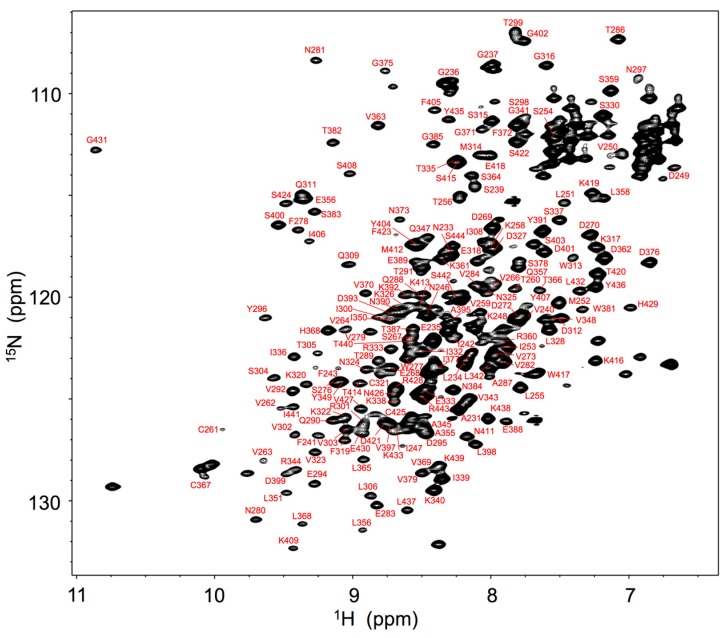
1H-15N HSQC spectrum of uniformly 13C, 15N-labeled mouse IgG2b-Fc recorded at 52 °C at 800 MHz. Backbone assignments are annotated by the resonance peaks with one-letter amino acid codes and the sequence.

For expression of stable isotope-labeled IgG glycoprotein, antibody-producing hybridoma cells were cultivated in a serum-free medium in which metabolic precursors were all labeled with ^13^C- and ^15^N-labeled analogs. The spectral assignments were made for the backbone of Fc fragment proteolytically cleaved from IgG based on a series of triple-resonance spectral data supplemented with spectral data obtained by amino acid-selective labeling. The ^1^H-^15^N heteronuclear single-quantum coherence (HSQC) peaks originating from the Fc backbone (Figure 1) were used for probing microenvironments surrounding the individual amino acid residues of Fc in serum.

**Figure 2:**
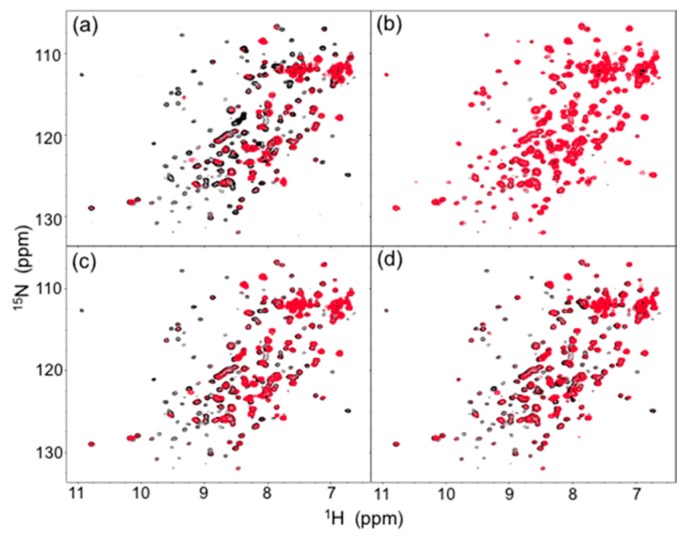
Comparison of ^1^H-^15^N HSQC spectra (red) of uniformly ^13^C, ^15^N-labeled mouse IgG2b-Fc (**a**) in human serum and in the presence of (**b**) HSA, (**c**) human polyclonal IgG, and (**d**) the Fab fragment derived from human serum polyclonal IgG with the spectrum of the mouse IgG2b-Fc dissolved in 5 mM sodium phosphate buffer, pH 7.4, containing 150 mM NaCl. The spectra were recorded at 37 °C at 800 MHz.

**Figure 3:**
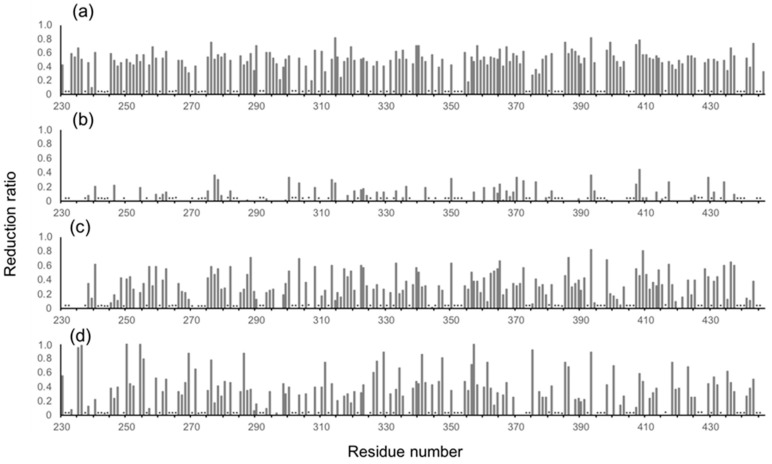
Profiles of the observed spectral perturbations by (**a**) human serum, (**b**) HSA, (**c**) human polyclonal IgG, and (**d**) the Fab fragment derived from human serum polyclonal IgG. The attenuation in intensity ((*I*o − *I*p)/*I*o, where *I*o and *I*p are original peak intensity and intensity after perturbation, respectively) of the HSQC cross peaks is plotted across the amino acid sequence of mouse IgG-Fc. Asterisks indicate proline residues, unassigned residues and residues whose peak intensity data could not be obtained due to severe peak overlapping.

In human serum, the isotope-labeled Fc exhibited significant reduction in intensity for many peaks, although the chemical shift of the residual peaks remained unchanged. The observed attenuations of peak intensity were quantified and mapped onto the crystal structure of Fc. The result indicated that most of the residues located on the Fc surface exhibited HSQC peak attenuation at varying magnitudes (Figure 2a and Figure 3a). Viscosity of the human serum was 1.177 ± 0.001 mPa·s (mean ± SD, *n* = 3), which was significantly higher than that of the control buffer (0.772 ± 0.001 mPa·s), and could slow down the tumbling of Fc, resulting in the peak intensity attenuation. However, the reductions in peak intensity were observed in a non-uniform manner, suggesting that the spectral perturbations were not simply because of the slowing down of molecular tumbling in the viscous serum environment, but were caused by interactions with serum components, as is the case with a variety of protein interaction systems [23].

The major proteins in human serum are IgG (10–20 mg/mL) and albumin (35–50 mg/mL). Therefore, we examined possible interactions of these serum proteins with isotope-labeled Fc fragments on the basis of HSQC spectral observations. Human serum albumin (HSA) caused only limited change in the spectrum of isotope-labeled Fc, while the addition of human polyclonal IgG evoked spectral changes similar to those observed in serum (Figure 2b,c and Figure 3b,c). Viscosities of the solutions of polyclonal IgG and HSA used in the present study were virtually identical, i.e. 0.866 ± 0.001 mPa·s and 0.850 ± 0.001 mPa·s for polyclonal IgG and HSA, respectively.

Therefore, the spectral changes were largely ascribed to specific or semi-specific interactions with serum polyclonal IgG. Moreover, the observed spectral perturbations were reproduced by the addition of the Fab fragment that was cleaved from polyclonal IgG fractions from the human serum (Figure 2d and Figure 3d). On the basis of these data, we conclude that serum-induced spectral perturbations of Fc are largely ascribed to its interactions with the Fab portions of the polyclonal IgG component.

**Figure 4:**
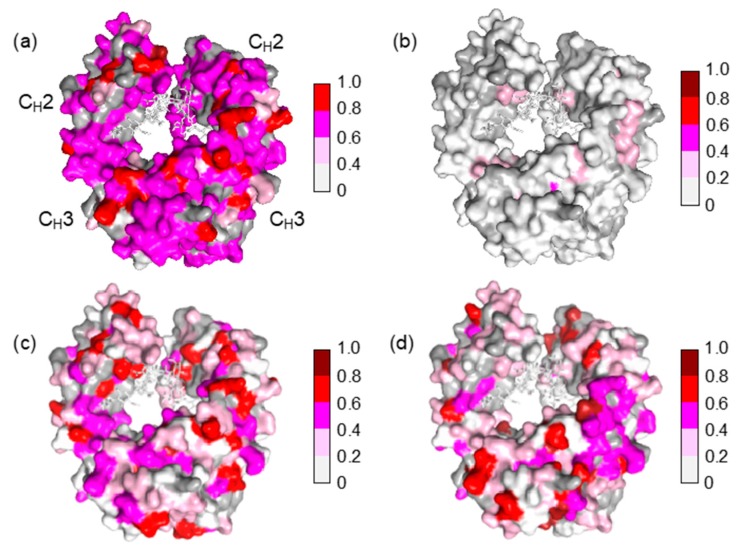
Mapping of the crystal structure of mouse IgG2b-Fc (PDB code: 2rgs) [30] with the observed spectral perturbations by addition of (**a**) human serum; (**b**) HAS; (**c**) human polyclonal IgG; and (**d**) the Fab fragment derived from human serum polyclonal IgG. The attenuation in intensity of the HSQC cross peaks is calculated as described in Figure 3. The proline residues and the residues whose ^1^H-^15^N HSQC peaks could not be observed as probe because of broadening and/or overlapping are shown in gray. The N-glycans are shown as stick models. The molecular graphics were generated using PyMOL [31].

The Fc fragment used in the present study was derived from mouse monoclonal IgG2b, which is a heterologous entity when present in human serum. It is intriguing that mouse IgG-Fc is potentially reactive with a subset of IgG antibodies that exists in human serum without preimmunization. The present NMR data indicate that the potential epitopes recognized by serum polyclonal IgG cover the entire molecular surface of Fc, including the binding sites for FcRn at the C_H_2-C_H_3 interface, FcγR at the hinge-proximal C_H_2 segments, and C1q at the C_H_2 surface [24,25,26,27,28,29] (Figure 4).

Our stable isotope labeling technique has thus enabled selective observation of NMR signals originating from an Fc glycoprotein in the heterogeneous serum environment, thereby opening the door to characterize *metaepitopes* recognized by polyclonal antibodies in the immune system. It is possible that engineered antibodies and recombinant antibody derivatives currently developed for pharmaceutical applications are involved in interaction networks with endogenous polyclonal antibodies. These interaction networks are transformable during immune processes, with the transformation contingent on physiological and pathological conditions, and are likely to influence the expected interactions of biopharmaceuticals with antigens and immune effector molecules. In the present study, we successfully established a technical basis for an *in-serum* NMR observation, which will offer useful tools for sensitive detection and detailed characterization of potential interactions of monoclonal antibodies and other recombinant glycoproteins as biopharmaceuticals in physiologically relevant heterogeneous environments. Furthermore, this line of study will provide deeper insights into molecular recognition by polyclonal antibodies in the immune system.

## 3. Materials and Methods

### 3.1. Human Serum

Pooled off-the-clot human serum was purchased from Access Biologicals (Vista, CA, USA). Polyclonal IgG was purified from 1 L of human serum through several consecutive steps. The initial purification step was precipitation with 40–60% saturated ammonium sulfate. The precipitate was re-solubilized in phosphate-buffered saline and then applied to a Blue Sepharose 6 Fast Flow column (GE Healthcare, Chicago, IL, USA) to remove albumin. The follow-through fraction was applied to a Protein G Sepharose 4 Fast Flow column (GE Healthcare) and then to a Superose 12 10/300 GL Chromatographic Separation Column (GE Healthcare). The Fab fragment of the polyclonal IgG was digested using papain and purified through a Protein G Sepharose column to remove Fc fragments, followed by applying it to a Superose 12 HR/20/30 gel-filtration column according to the literature [32]. Human serum IgG and HSA were purchased from Sigma-Aldrich (St. Louis, MO, USA).

### 3.2. Preparation of Isotope-Labeled Fc

Metabolic isotope labeling of antibody was performed according to a previously reported protocol [19]. Briefly, we used monoclonal mouse anti-progesterone IgG2b, which was produced using the hybridoma cell line 7D7 [33] grown in a modified Nissui NYSF-404 medium containing the appropriate stable isotope-labeled metabolic precursors. For amino acid-selective labeling, selected amino acids were substituted with ^13^C- and/or ^15^N-labeled analogs. For uniform labeling, metabolic carbon sources, i.e., glucose, sodium pyruvate, and succinic acid, were all labeled with ^13^C, and the amino acid components were replaced by ^13^C- and ^15^N-labeled algal amino acid mixture supplemented with ^13^C-/^15^N-labeled analogs of the following amino acids: l-leucine, l-histidine, l-glutamine, l-cysteine, and l-asparagine. The Fc fragment of mouse IgG2b was prepared through proteolytic digestion using papain (for spectral assignments) and V8 protease (for chemical shift perturbation experiments) as described previously [34] and subjected to NMR measurements.

### 3.3. Viscosity Measurements

The densities and viscosities of the samples were measured at 37 °C using a density meter DMA5000 (Anton Paar, Ashland, VA, USA) and a viscometer Lovis 2000ME (Anton Paar), respectively.

### 3.4. NMR Measurements and Spectral Analysis

For NMR measurements, the concentration of the ^15^N-labeled Fc fragment was set to 6 mg/mL. The concentration of polyclonal IgG and HSA were set to 30 mg/mL and 40 mg/mL, respectively, in 5 mM sodium phosphate buffer containing 50 mM NaCl. The pH and temperature of the solutions was set to pH 7.4 and 37 °C. ^1^H-^15^N HSQC peaks originating from the backbone of Fc dissolved in 5 mM sodium phosphate buffer, pH 6.0, containing 50 mM NaCl (at 52 °C) were assigned based on conventional triple-resonance and three-dimensional transverse relaxation optimized spectroscopy datasets of HNCO, HNCACO, HNCA, and HNCOCA, in conjunction with the amino acid-selective subspectral observations. A series of NMR spectra were measured using DRX-400, DMX-500, AVANCE 800 and AVANCEIII 900, and 950 spectrometers (Bruker BioSpin, Rheinstetten, Germany). The acquired data were processed using NMRpipe [35] and MagRO [36,37]. The assignments for the ^1^H, ^13^C, and ^15^N backbone resonances of human IgG1-Fc have been deposited in the BioMagResBank database (http://www.bmrb.wisc.edu) under the accession number 27208.

## References

[1] Jerne N.K. (1974). Towards a network theory of the immune system. Ann. D’immunologie.

[2] Malhotra R., Wormald M.R., Rudd P.M., Fischer P.B., Dwek R.A., Sim R.B. (1995). Glycosylation changes of Igg associated with rheumatoid-arthritis can activate complement via the mannose-binding protein. Nat. Med..

[3] Krayukhina E., Noda M., Ishii K., Maruno T., Wakabayashi H., Tada M., Suzuki T., Ishii-Watabe A., Kato M., Uchiyama S. (2017). Analytical ultracentrifugation with fluorescence detection system reveals differences in complex formation between recombinant human TNF and different biological TNF antagonists in various environments. Mabs-Austin.

[4] Reichert J.M. (2017). Antibodies to watch in 2017. Mabs-Austin.

[5] Dillman R.O., Shawler D.L., McCallister T.J., Halpern S.E. (1994). Human anti-mouse antibody response in cancer patients following single low-dose injections of radiolabeled murine monoclonal antibodies. Cancer Biother. Radiopharm..

[6] Ritter G., Cohen L.S., Williams C., Richards E.C., Old L.J., Welt S. (2001). Serological analysis of human anti-human antibody responses in colon cancer patients treated with repeated doses of humanized monoclonal antibody A33. Cancer Res..

[7] Hansel T.T., Kropshofer H., Singer T., Mitchell J.A., George A.J. (2010). The safety and side effects of monoclonal antibodies. Nat. Rev. Drug Discov..

[8] Narciso J.E., Uy I.D., Cabang A.B., Chavez J.F., Pablo J.L., Padilla-Concepcion G.P., Padlan E.A. (2011). Analysis of the antibody structure based on high-resolution crystallographic studies. New Biotechnol..

[9] Sela-Culang I., Kunik V., Ofran Y. (2013). The structural basis of antibody-antigen recognition. Front. Immunol..

[10] Kato K., Yamaguchi Y., Harris R.K., Wasylishen R.E. (2011). Glycoproteins and Antibodies: Solution NMR Studies. Encyclopedia of Magnetic Resonance.

[11] Inomata K., Ohno A., Tochio H., Isogai S., Tenno T., Nakase I., Takeuchi T., Futaki S., Ito Y., Hiroaki H. (2009). High-resolution multi-dimensional NMR spectroscopy of proteins in human cells. Nature.

[12] Theillet F.X., Binolfi A., Bekei B., Martorana A., Rose H.M., Stuiver M., Verzini S., Lorenz D., van Rossum M., Goldfarb D. (2016). Structural disorder of monomeric α-synuclein persists in mammalian cells. Nature.

[13] Anderson N.L., Anderson N.G. (2002). The human plasma proteome: history, character, and diagnostic prospects. Mol. Cell. Proteom..

[14] Solá R.J., Griebenow K. (2010). Glycosylation of therapeutic proteins: An effective strategy to optimize efficacy. BioDrugs Clin. Immunother. Biopharma. Gene Ther..

[15] Solá R.J., Griebenow K. (2009). Effects of glycosylation on the stability of protein pharmaceuticals. J. Pharm. Sci..

[16] Mimura Y., Katoh T., Saldova R., O’Flaherty R., Izumi T., Mimura-Kimura Y., Utsunomiya T., Mizukami Y., Yamamoto K., Matsumoto T. (2017). Glycosylation engineering of therapeutic IgG antibodies: Challenges for the safety, functionality and efficacy. Protein Cell.

[17] Kato K., Yamaguchi Y., Arata Y. (2010). Stable-isotope-assisted NMR approaches to glycoproteins using immunoglobulin G as a model system. Prog. Nucl. Magn. Reson. Spectrosc..

[18] Yagi H., Zhang Y., Yagi-Utsumi M., Yamaguchi T., Iida S., Yamaguchi Y., Kato K. (2015). Backbone ^1^H, ^13^C, and ^15^N resonance assignments of the Fc fragment of human immunoglobulin G glycoprotein. Biomol. NMR Assign..

[19] Yamaguchi Y., Kato K. (2010). Dynamics and interactions of glycoconjugates probed by stable-isotope-assisted NMR spectroscopy. Methods Enzymol..

[20] Yagi H., Fukuzawa N., Tasaka Y., Matsuo K., Zhang Y., Yamaguchi T., Kondo S., Nakazawa S., Hashii N., Kawasaki N. (2015). NMR-based structural validation of therapeutic antibody produced in *Nicotiana benthamiana*. Plant Cell Rep..

[21] Yamaguchi Y., Yagi H., Kato K., Kato K., Peters T. (2017). Stable isotope labeling of glycoproteins for NMR study. NMR in Glycoscience and Glycotechnology.

[22] Yagi H., Nakamura M., Yokoyama J., Zhang Y., Yamaguchi T., Kondo S., Kobayashi J., Kato T., Park E.Y., Nakazawa S. (2015). Stable isotope labeling of glycoprotein expressed in silkworms using immunoglobulin G as a test molecule. J. Biomol. NMR.

[23] Driscoll P.C., Lian L., Roberts G. (2011). Macrimolecular Complexes. Protein NMR Spectroscopy: Principal Techniques and Applications.

[24] Idusogie E.E., Presta L.G., Gazzano-Santoro H., Totpal K., Wong P.Y., Ultsch M., Meng Y.G., Mulkerrin M.G. (2000). Mapping of the C1q binding site on rituxan, a chimeric antibody with a human IgG1 Fc. J. Immunol..

[25] Mizushima T., Yagi H., Takemoto E., Shibata-Koyama M., Isoda Y., Iida S., Masuda K., Satoh M., Kato K. (2011). Structural basis for improved efficacy of therapeutic antibodies on defucosylation of their Fc glycans. Genes Cells.

[26] Kiyoshi M., Caaveiro J.M., Kawai T., Tashiro S., Ide T., Asaoka Y., Hatayama K., Tsumoto K. (2015). Structural basis for binding of human IgG1 to its high-affinity human receptor FcγRI. Nat. Commun..

[27] Martin W.L., West A.P., Gan L., Bjorkman P.J. (2001). Crystal structure at 2.8 Å of an FcRn/heterodimeric Fc complex: Mechanism of pH-dependent binding. Mol. Cell.

[28] Ferrara C., Grau S., Jäger C., Sondermann P., Brünker P., Waldhauer I., Hennig M., Ruf A., Rufer A.C., Stihle M. (2011). Unique carbohydrate-carbohydrate interactions are required for high affinity binding between FcγRIII and antibodies lacking core fucose. Proc. Natl. Acad. Sci. USA.

[29] Sun P., Ackerman M.E., Nimmerjahn F. (2013). Structural Recognition of Immunoglobulins by Fcγ Receptors. Antibody Fc: Linking Adaptive and Innate Immunity.

[30] Kolenko P., Dohnálek J., Dusková J., Skálová T., Collard R., Hasek J. (2009). New insights into intra- and intermolecular interactions of immunoglobulins: Crystal structure of mouse IgG2b-Fc at 2.1-Å resolution. Immunology.

[31] DeLano W.L. (2002). The PyMOL Molecular Graphics System.

[32] Yagi H., Takahashi N., Yamaguchi Y., Kato K. (2004). Temperature-dependent isologous Fab-Fab interaction that mediates cryocrystallization of a monoclonal immunoglobulin G. Mol. Immunol..

[33] Sawada J., Terao T., Itoh S., Maeda M., Tsuji A., Hosoda H., Nambara T. (1987). Production and characterization of monoclonal antibodies to 17 α-hydroxyprogesterone. J. Steroid Biochem..

[34] Yamaguchi Y., Kim H., Kato K., Masuda K., Shimada I., Arata Y. (1995). Proteolytic fragmentation with high specificity of mouse immunoglobulin G. Mapping of proteolytic cleavage sites in the hinge region. J. Immunol. Methods.

[35] Delaglio F., Grzesiek S., Vuister G.W., Zhu G., Pfeifer J., Bax A. (1995). Nmrpipe—A multidimensional spectral processing system based on Unix Pipes. J. Biomol. NMR.

[36] Kobayashi N., Iwahara J., Koshiba S., Tomizawa T., Tochio N., Güntert P., Kigawa T., Yokoyama S. (2007). KUJIRA, a package of integrated modules for systematic and interactive analysis of NMR data directed to high-throughput NMR structure studies. J. Biomol. NMR.

[37] Kobayashi N., Harano Y., Tochio N., Nakatani E., Kigawa T., Yokoyama S., Mading S., Ulrich E.L., Markley J.L., Akutsu H. (2012). An automated system designed for large scale NMR data deposition and annotation: Application to over 600 assigned chemical shift data entries to the BioMagResBank from the Riken Structural Genomics/Proteomics Initiative internal database. J. Biomol. NMR.

